# An Unusual Cause of Transient Ischemic Attack in a Patient with Pacemaker

**DOI:** 10.1155/2014/265759

**Published:** 2014-02-04

**Authors:** Jagadeesh Kumar Kalavakunta, Vishal Gupta, Basil Paulus, William Lapenna

**Affiliations:** ^1^Division of Cardiology, Michigan State University, 804 Service Road, A205 Clinical Center, East Lansing, MI, 48824, USA; ^2^Division of Cardiology, Borgess Medical Center, Kalamazoo, MI 49048, USA

## Abstract

Pacemaker lead malposition in various locations has been described in the literature. Lead malposition in left ventricle is a rare and an underdiagnosed complication. We present a 77-year-old man with history of atrial fibrillation and pacemaker placement who was admitted for transient ischemic attack. He was on aspirin, beta blocker, and warfarin with subtherapeutic international normalized ratio. His paced electrocardiogram showed right bundle-branch block, rather than the typical pattern of left bundle-branch block, suggesting pacemaker lead malposition. Further, his chest X-ray and echocardiogram confirmed the pacemaker lead position in the left ventricle instead of right ventricle. He refused surgical removal of the lead and we increased his warfarin dose. Diagnosis of lead malposition in left ventricle, though easy to identify in echocardiogram, requires high index of clinical suspicion. In asymptomatic patients, surgical removal may be deferred for treatment with lifelong anticoagulation.

## 1. Case Presentation

**Figure 1:**
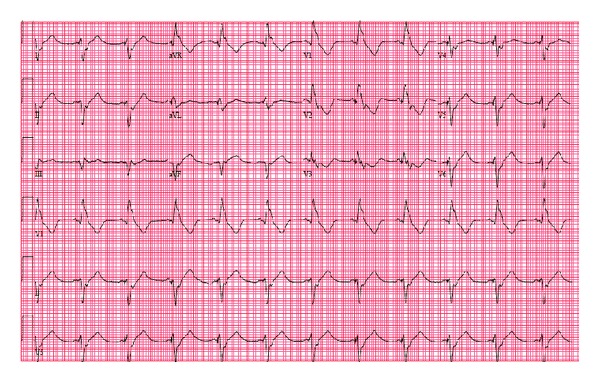
A 12-lead electrocardiogram (ECG) showing (right bundle-branch block RBBB) morphology.

**Figure 2:**
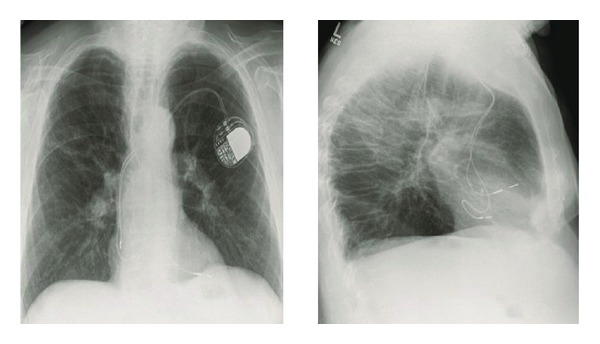
Chest radiograph PA and lateral projection showing the ventricular lead with an abnormal configuration.

**Figure 3:**
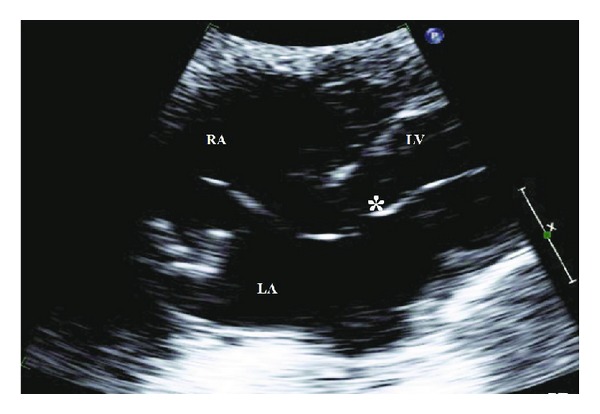
Transthoracic echocardiography, subcostal view showing the ventricular pacing lead (∗) to pass from the right atrium via interatrial septal defect to the left atrium and then via the mitral valve to the left ventricle. RA: right atrium; LA: left atrium; LV: left ventricle.

We present a 77-year-old Caucasian man with history of multiple comorbidities including coronary artery disease, diabetes mellitus, atrial fibrillation, pacemaker placement, and 40-pack-years of smoking who presented with complaints of speech disturbance and left-sided numbness and tingling which resolved within couple of hours of the hospitalization. He was diagnosed with transient ischemic attack (TIA). His pacemaker had been implanted for tachy-brady syndrome 36 months prior to this presentation. He was on warfarin, aspirin, and beta blocker for atrial fibrillation. His pulse rate was 105 beats/min, blood pressure 121/51 mmHg, respiratory rate 20/min, and oxygen saturation 96% on 3 L of oxygen. Physical examination revealed irregularly irregular tachycardia, bilateral rhonchi, wheezes, and mild pedal edema. His international normalized ratio (INR) was subtherapeutic at 1.2. Computer tomographic scan of the head was negative for acute process. His electrocardiogram (ECG) showed paced rhythm with right bundle-branch block, rather than the typical pattern of left bundle-branch block (Figure 1). His chest X-ray in lateral projection showed ventricular lead with an abnormal turn (Figure 2). Given the abnormal ECG and chest X-ray findings, pacemaker lead malposition was suspected. Transthoracic echocardiogram confirmed the pacemaker lead position in the left ventricle apex instead of right ventricle, passing through the interatrial septal defect (Figure 3). Cardiac chambers were nondilated with ejection fraction of 65%. There was no associated thrombus. Doppler color-flow showed normal peak flows along with mild mitral regurgitation. Carotid ultrasound did not reveal any significant stenosis. Neurology service was on board and did the standard workup for TIA/stroke, which was negative.

We explained the possibility of recurrence of stroke/TIA in the setting of the malpositioned pacemaker lead to him. In view of his multiple co-morbidities, he refused surgical removal of the lead or any invasive procedures, including transesophageal echocardiogram. We increased his warfarin dose and educated him to maintain his INR between 2.5 and 3.5. Two years after the clinical follow-up, there was no evidence of further thromboembolic phenomenon.

## 2. Discussion

In the United States of America, more than 200,000 permanent cardiac pacemakers are implanted annually [1]. Pacemaker lead malposition in variety of locations has been described in the literature. Lead malposition in left ventricle (LV) is rare and an underdiagnosed complication. A number of anatomic routes for malposition have been described, including patent foramen ovale, atrial septal defect, sinus venosus type defects, or perforations through the inter-atrial septum, atrioventricular membrane, interventricular membrane, and rarely through right ventricle apex [2–5].

The natural history of the lead malposition in the LV is not well understood, due to underreporting and the lack of long-term follow-up reports. In most cases, patients are asymptomatic, but severe complications such as embolic strokes and TIAs can occur. The incidence of the thromboembolic complications is not known. Van Gelder et al. suggested that up to 37% of patients may experience them [6]. Other complications include mitral and aortic valve damage, endocarditis, diaphragmatic pacing, and loss of capture [7, 8]. In this case, our patient had symptoms of TIA. We hypothesized that the malposition of the pacemaker lead was the culprit and evaluated him further.

Diagnosis of lead malposition in left ventricle, though easy to detect in echocardiogram, requires high index of clinical suspicion. Chest X-ray and paced ECG may suggest lead malposition. On ECG, right ventricular (RV) pacing will show left bundle-branch block (LBBB) and left ventricular pacing will show right bundle-branch block (RBBB). Okmen and his colleagues proposed criteria with high sensitivity and specificity to differentiate RBBB pattern during RV and LV pacing based on (1) left superior axis deviation in the frontal plane between −30° and −90°, (2) precordial transition at V3, (3) qR or RS in V1 precordial lead, and (4) the absence of S wave in lead I [9].

In general, the malposition is missed after pacemaker placement because only limited leads are used to interrogate the pacemaker, and limited fluoroscopy views are used during the procedure. For example, it is difficult to differentiate which ventricle the lead enters on the commonly utilized right anterior oblique (RAO) view, as the ventricles are visualized from one side only. Malposition can be identified immediately by performing a 12-lead ECG with ventricular capture after procedure and applying Okmen's criteria. Additional fluoroscopy views, such as left anterior oblique (LAO), should be used whenever there is doubt regarding lead placement.

In our patient, the chest X-ray suggested abnormal positioning of the lead and his ECG showed RBBB. The transthoracic echocardiogram confirmed malposition, showing the lead traversing an interatrial septal defect to the left atrium and the mitral valve before lodging at the apex of the left ventricle.

Surgical removal of the malpositioned lead should be contemplated in all patients with symptoms [10]. Percutaneous removal or laser techniques are contraindicated due to procedural-related dislodgement of thrombi into systemic circulation [6]. In asymptomatic patients, surgical removal can be deferred and the patient started on lifelong anticoagulation, maintaining the INR >2.5 [11]. Our patient was on warfarin and aspirin already, although his INR had become subtherapeutic. After reviewing the risks and benefits of intervention, our patient refused surgical removal. We increased his warfarin dosage to maintain anticoagulation in the therapeutic range. In the literature, there are no recurrences of thromboembolic phenomenon after the surgical removal of the pacemaker lead or on chronic anticoagulation with adequate INR. After two years, our patient had not had any recurrent symptoms either.
